# Augmented reality-assisted infraclavicular first rib resection for arterial and venous thoracic outlet syndrome: a case series

**DOI:** 10.1186/s13019-025-03665-7

**Published:** 2025-10-22

**Authors:** Ryogo Furuhata, Atsushi Tanji, Yuki Yamai, Taku Suzuki

**Affiliations:** 1 Department of Orthopaedic Surgery, Ashikaga Red Cross Hospital, 284-1 Yobe-cho, Ashikaga, 326-0843 Tochigi Japan; 2 Department of Orthopaedic Surgery, Keio University School of Medicine, Shinjuku-ku, Tokyo, Japan

**Keywords:** Thoracic outlet syndrome, First rib resection, Infraclavicular approach, Augmented reality, Outcome

## Abstract

**Background:**

The infraclavicular approach is a surgical approach for vascular thoracic outlet syndrome (TOS). However, difficulty in accessing the posterior aspect of the first rib may cause insufficient decompression. To address this problem, we used augmented reality (AR) technology to intraoperatively visualize and determine the extent of rib resection. This study aimed to introduce AR-assisted infraclavicular first rib resection for arterial or venous TOS and report its clinical outcomes.

**Methods:**

AR-assisted rib resection was introduced in our unit in 2022. Using an infraclavicular approach, we performed first rib resection and scalenectomy with the assistance of endoscopy. We compared the edge of the intraoperatively resected rib with the resection area of the preoperative simulation displayed in the AR to determine the extent of the resection.

**Results:**

Six patients who underwent AR-assisted rib resection for arterial or venous TOS had excellent or good Derkash scores at 1 year postoperatively. Postoperative angiography revealed no subclavian vessel stenosis.

**Conclusions:**

AR technology enables intraoperative three-dimensional assessment of the location of major vessels and the extent of resection, which may contribute to improved outcomes.

## Introduction

Vascular thoracic outlet syndrome (TOS) is a constellation of symptoms caused by the compression of subclavian vessels. It includes arterial and venous TOS, which are exceedingly rare, occurring in 1–2% and 3–16% of all TOS cases, respectively [1–3].

The infraclavicular approach is a major surgical approach for vascular TOS; however, surgical access to the posterior aspects of the first rib is difficult, suggesting insufficient decompression of the posterior components [4, 5]. Incomplete resection can cause poor postoperative outcomes [6]. Previous clinical studies have reported that infraclavicular first rib resection (IC-FRR) for venous TOS achieves a high postoperative venous primary patency rate (84–100%) [7–12] and satisfactory functional outcomes; however, recurrences due to stenosis from inadequate resection of the posterior first rib have also been documented [13].

To address this problem, we used augmented reality (AR) technology to intraoperatively visualize and determine the extent of resection. AR is a computer vision technology that enables model three-dimensional (3D) images to be superimposed on actual objects in real time [14, 15]. The application thereof in surgery enables 3D visualization and interaction with the surgical field [15].

AR technology has been used in total elbow arthroplasty [15] and spine surgery [14]. In total elbow arthroplasty, the application of our developed AR system resulted in more accurate component placement than the conventional technique in a cadaver study [6]. AR-assisted total elbow arthroplasty demonstrated both translational and rotational errors of the humeral component within the clinically acceptable range (< 2 mm for translation; < 2º for rotation) [6]. However, there are no reports of the application to TOS surgery. We, therefore, aimed to introduce and report the postoperative outcomes of AR-assisted IC-FRR for arterial or venous TOS.

## Methods

### Preoperative simulation

For preoperative simulation, computed tomography (CT) images with a slice thickness of 1.0 mm were obtained from the neck to the chest with the upper extremities positioned at the sides. Surface geometry models of the clavicle, ribs, subclavian artery, and veins were created in a computed tomography workstation (Vincent; Toshiba, Kanagawa, Japan) using a marching cube algorithm [16]. Surface geometry models were generated for each patient based on their individual CT imaging data. We incorporated these surface models into our simulation software (Mac OS X operating system and Xcode integrated development environment; Apple, Cupertino, CA). The software used in this study was developed by one of the authors (Atsushi Tanji). The appropriate resection area was set at a 2-cm margin from the subclavian vessels [5], and the resection was simulated. This produced a surface model of the first rib after appropriate resection. The coordinates of three points on the first rib (a posterior point of the sternocostal joint and anterior and posterior points of the first rib, 3 cm proximal to the sternocostal joint) were also recorded on the simulator. During surgery, registration was performed by pointing to the pre-set areas. These data were inputted into a dedicated file format and loaded onto an AR application on a mobile phone.

### Surgical procedure

**Fig. 1:**
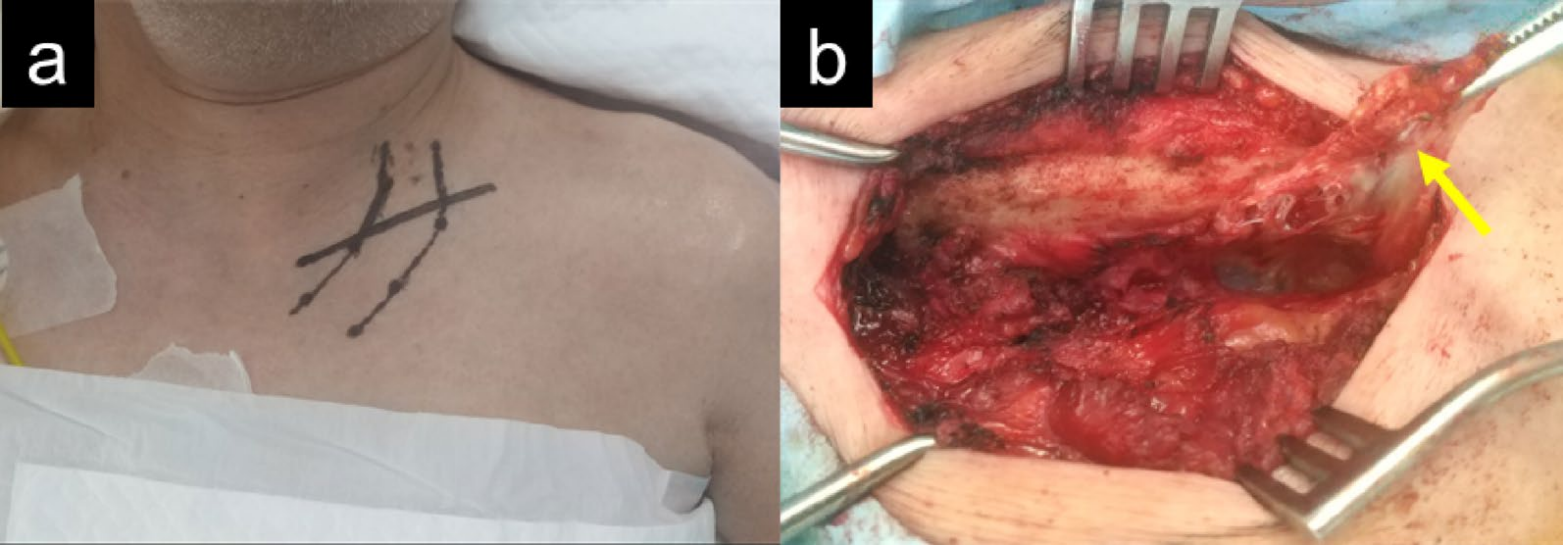
Intraoperative images of the infraclavicular approach. A transverse incision is made 1 cm below the clavicle (**a**). Image after resection of the subclavian muscle (yellow arrow), the first rib (**b**)

Surgery was performed in the beach chair position under general anesthesia. An 8-cm transverse incision was made below the clavicle (Fig. 1a). The pectoralis major muscle was dissected from the clavicle. After the resection of the subclavian muscle (Fig. 1b), the first rib was exposed.

**Fig. 2:**
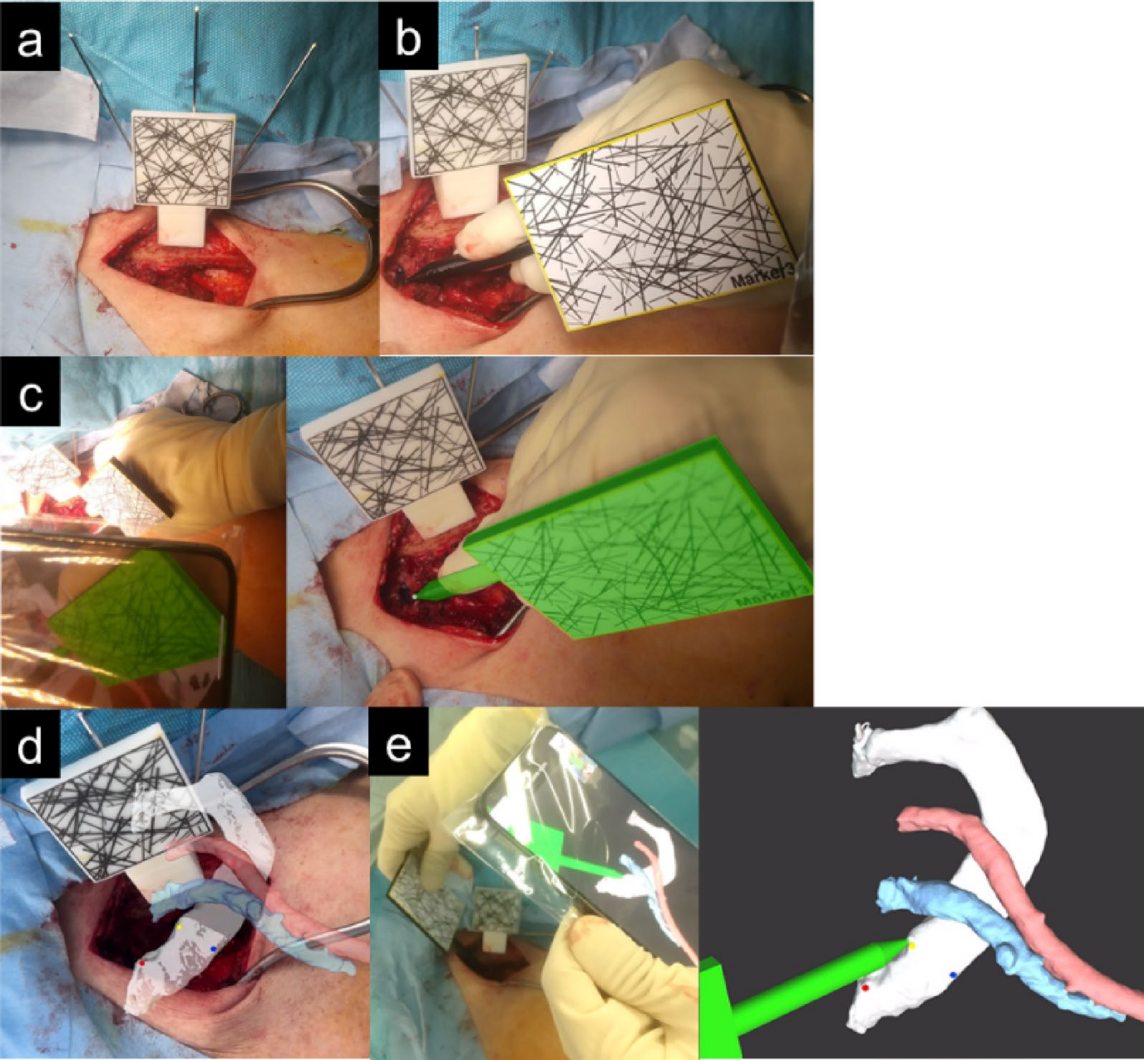
Augmented reality (AR) setup. A block with AR markers is placed on the clavicle by inserting three Kirschner wires through the holes (**a**). A pointer with an AR marker (**b**). Registration by touching three points on the first rib and sternum with this marker while reading the AR markers using the camera on a mobile phone. When the AR marker was recognized by the mobile phone camera, the pointer was displayed in green (left panel: intraoperative view, right panel: mobile phone view) (**c**). Surface models of the first rib (white), subclavian artery (red), and vein (blue) displayed in AR (mobile phone view) after completion of registration (**d**). We confirmed that the surface model overlapped with the shape of the first rib (left panel: intraoperative view, right panel: mobile phone view) (**e**)

The equipment required for the AR setup included an AR marker, a block, a pointer, a mobile phone with the AR application installed, three 1.5 mm Kirschner wires, and a power tool. The AR marker was used to obtain positional information by capturing images through the mobile phone camera while the AR application was running. Initially, a block, a rectangular device with multiple Kirschner wire holes, was fixed to the clavicle using three Kirschner wires, and an AR marker was attached on top of the block to provide positional information on the position of the clavicle during registration (Fig. 2a). With the AR application running, we used a pointer for registration. This pointer was a stylus pen-shaped device equipped with an AR marker (Fig. 2b). Before registration, we superimposed the pointer’s surface model onto the actual pointer and confirmed that they were completely aligned. If there was any error, we input the error information into the AR application and corrected it to calibrate. When the AR marker on this device was detected by the mobile phone camera, the position of the pointer tip was displayed on the mobile phone screen. We performed registration by touching three points on the first rib with a pointer while reading the AR markers with a mobile phone camera (Fig. 2c). We captured the surgical field using a camera, and the image in the field was superimposed on the surface model of the first rib and subclavian vessels (Fig. 2d). We overlaid the first rib’s surface model and confirmed that it accurately aligns with the actual first rib. Using the pointer, we indicated the corresponding location on the actual rib and verify that it matched the indicated location on the superimposed rib. These verification steps confirmed that registration was performed accurately (Fig. 2e). In the presented case, 4 min and 21 s were required to complete the AR setup. No unexpected delays or technical issues with the AR setup were observed in the remaining cases.

**Fig. 3:**
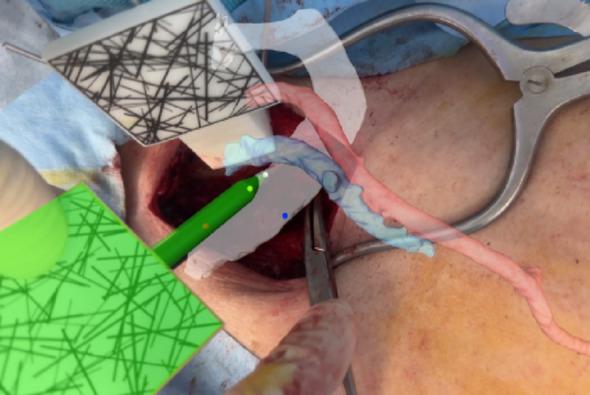
Exposure of subclavian vein. With the location of the subclavian vein displayed in the augmented reality, the subclavian vein was identified and exposed in an actual surgical field (mobile phone view)

AR technology was utilized during the exposure of the subclavian vein and the assessment of the extent of first rib resection. During these phases, the surgical procedure was performed while observing the operative field through a mobile phone camera. A real-time image of the operative field was displayed on the mobile device, with AR-based surface models of the first rib and subclavian vessels superimposed. Using the subclavian vein location displayed in the AR, we identified and exposed the subclavian vein in the actual surgical field (Fig. 3).

**Fig. 4:**
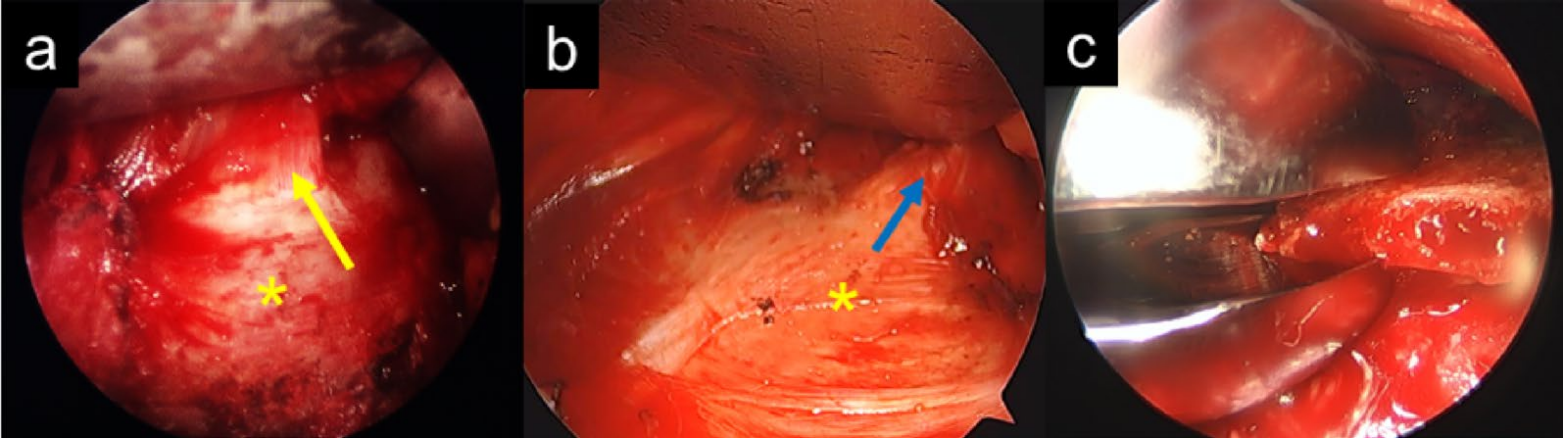
Endoscopic images of the first rib resection and scalenectomy. Resection of the anterior scalene muscle (yellow arrow) from the origin of the first rib (asterisk) (**a**). Using a 30° oblique arthroscope through the same incision, the first rib (asterisk) is resected up to the attachment of the middle scalene muscle (blue arrow) (**b**). Under endoscopic view, we resected the first rib piece-by-piece using a Luer rongeur (**c**)

According to the procedure described by Suzuki et al. [17], we inserted a 30° oblique arthroscope through the same incision to perform scalenectomy (Fig. 4a and b) and first rib resection (FRR) (Fig. 4c).

Resection of the anterior scalene muscle (yellow arrow) from the origin of the first rib (asterisk) (**a**). Using a 30° oblique arthroscope through the same incision, the first rib (asterisk) is resected up to the attachment of the middle scalene muscle (blue arrow) (**b**). Under endoscopic view, we resected the first rib piece-by-piece using a Luer rongeur (**c**)

**Fig. 5:**
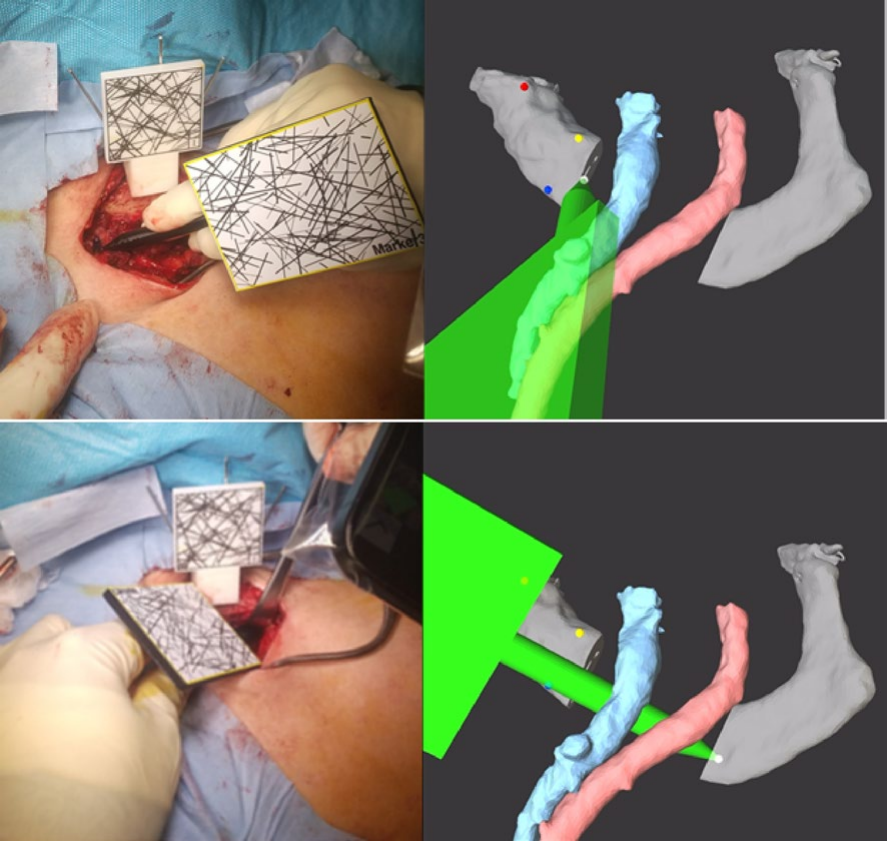
Determination of the extent of resection of the first rib. During first rib resection, we traced the anterior and posterior margins of the resected first rib with a pointer and compared them with the preoperatively planned extent of resection (left panel: intraoperative view; right panel: mobile phone view)

To determine the extent of intraoperative first rib resection, arthroscopic surgery was temporarily suspended, and AR technology was utilized. We traced the edge of the resected rib with a pointer and compared it with the resection area of the preoperative simulation displayed in the AR to determine the extent of the intraoperative resection (Fig. 5).

### Patients

Between 2022 and 2023, AR-assisted IC-FRR was performed on all patients diagnosed with TOS who had symptoms that interfered with daily life and who desired surgery. We included patients who met al.l of the following criteria: (1) those who underwent AR-assisted IC-FRR between 2022 and 2023 and could be followed up for more than 1 year; (2) with a positive Wright test (a decrease in the radial pulse in abduction and external rotation position of shoulder joint) [18]; (3) with stenosis of the subclavian artery or vein at the costoclavicular space confirmed in CT or magnetic resonance angiography with upper extremity elevation.

Patients were excluded if they met any of the following criteria: (1) a positive Morley test, defined by tenderness and a Tinel sign in the supraclavicular fossa [19], suggestive of neurogenic TOS; (2) a requirement for extensive venous reconstruction [5]; or (3) TOS associated with a cervical rib or short first rib hypoplasia [20], which remains a contraindication for the infraclavicular approach despite recent advancements in endoscopic techniques.

### Outcome measures

We evaluated the postoperative functional outcomes using the Derkash score (a four-point scale (excellent, good, fair, or poor) used to evaluate functional outcomes in TOS surgery) [21]; disability of the arm, shoulder, and hand (DASH) questionnaire score (patient-reported score in upper limb musculoskeletal disorders) [22]; and grip strength at 1 year postoperatively. We also evaluated stenosis of the subclavian vessels in CT angiography within 1 year postoperatively.

## Results

We identified six patients who met the inclusion criteria (Table 1). Two patients had Paget–Schroetter syndrome and received heparin for 2 weeks preoperatively and anticoagulation medication for 6 months postoperatively.

**Table 1:** Details of patients who underwent augmented reality-assisted first rib resection for vascular thoracic outlet syndrome

Age/sex	Stenosis		PSS	Operative time (min)	Blood loss (g)	Derkash score	DASH score		Grip strength (kg)		Postoperative stenosis	Complication
	Artery	Vein					Pre	Post	Pre	Post		
23/Male	+	+	-	211	78	Excellent	39	3	13	42	No data	-
68/Male	+	+	-	148	24	Excellent	60	10	15	28	-	-
43/Male	+	+	+	176	67	Excellent	55	7.5	40	43	-	Hemothorax
46/Female	+	+	-	125	79	Excellent	45	8	23	26	-	-
45/Male	+	+	+	179	21	Good	58	37	15	20	-	-
38/Female	+	+	-	151	31	Good	55	30	13	18	-	-

PSS, Paget–Schroetter syndrome; DASH, Disabilities of the arm, shoulder, and hand; Pre, Preoperative; Post, Postoperative

Regarding perioperative outcomes, the mean operative time was 165 ± 27 min; the mean intraoperative blood loss was 50 ± 15 g, and the mean postoperative length of hospital stay was 6 ± 4 days. The mean visual analogue scale score at one day postoperatively was 5.5 ± 5.6. Postoperative drains were not inserted in any case except one, which was complicated by hemothorax.

The postoperative Derkash score was excellent in four patients and good in two patients. All patients showed improvements in the DASH score and grip strength. The DASH score decreased from 52± 7 preoperatively to 16 ± 13 1-year postoperatively, and grip strength increased from 20 ± 10 kg preoperatively to 30 ± 10 kg 1-year postoperatively. CT angiography showed no stenosis in the subclavian vessels, and thrombus in the subclavian vein disappeared in the patient with a preoperative thrombus.

## Discussion

We introduced AR-assisted IC-FRR for arterial and venous TOS. AR-assisted FRR yields satisfactory postoperative functional outcomes and subclavian vessel decompression, which are comparable to the results of previous reports of IC-FRR [7–12, 23].

Recently, Suzuki et al. reported that endoscopy enabled the visualization and resection of the posterior aspects of the first rib, which expanded the indication of IC-FRR to neurogenic TOS [17]. However, even with the use of endoscopy, it is difficult to determine the extent of rib resection under 3D visualization because the position of the end of the first rib and the neurovascular bundle is detected by two-dimensional endoscopy monitoring. Prior to the implementation of AR technology, multiple intraoperative radiographs were required at our institution to determine the extent of rib resection. Additionally, an intraoperative subclavian vein injury had occurred in one case, which was repaired by a vascular surgeon.

This technique offers two major advantages. First, the surgical field and superimposed preoperative vascular images are displayed on the same monitor, allowing the surgeon to intraoperatively locate the subclavian vessels hidden in the clavicle and soft tissues, reducing the risk of vascular injury, a potentially fatal complication. Second, the use of AR allows real-time 3D comparison of the extent of rib resection planned in the preoperative computer simulation with the edge of the resected rib during surgery, which can result in reliable decompression.

The disadvantages of AR technology include the cost of the setup and the potential increase in operative time associated with its use. The AR application and equipment used in this study were developed by the study’s author and are planned for future commercialization. The hospital cost for the required equipment is approximately $50 for three Kirschner wires; however, the final pricing for commercially manufactured or rented equipment has not yet been determined. Accurate evaluation of the impact of AR technology on operative time is limited, as this study lacks a comparison group that underwent IC-FRR without AR assistance. Although the AR setup time was approximately 5 min, previous studies on IC-FRR for venous TOS have reported mean operative times of 2–2.2 h [8, 10], which was shorter than that of our case series. Since endoscopic techniques were not employed in the previous studies [8, 10], the discrepancy in operative time may be attributed to endoscope use in the present series; however, the possibility that AR technology contributed to increased surgical time cannot be excluded [8, 10]. The learning curve could not be evaluated due to the limited number of cases; however, only the initial case exceeded 3 h, while subsequent procedures ranged from 2 h to 20 min to 3 h. This raises the possibility that the learning curve of AR-assisted first rib resection was relatively gradual.

In addition, the AR-assisted infraclavicular approach used in this study demonstrated specific advantages and disadvantages compared to other surgical approaches for vascular TOS. The transaxillary and supraclavicular approaches, commonly utilized in TOS surgery, provide enhanced visualization of the posterior aspect of the first rib and the scalene muscles. However, compared to these approaches, the present approach allows for easier access to the anterior structure of the thoracic outlet, including the subclavian muscle, the anterior aspect of the first rib, and the costoclavicular ligament in a shallower surgical field [4, 5], which may offer advantages in vascular TOS. A recent meta-analysis reported that postoperative clinical improvement following the infraclavicular approach was significantly higher than that observed with the transaxillary approach in cases of venous TOS [24]. Another comparative study showed that the infraclavicular approach was associated with significantly lower postoperative complication rates and higher postoperative subclavian vein patency than the supraclavicular approach [11, 12]. Recently, minimally invasive techniques, such as video-assisted and robotic-assisted thoracoscopic surgery, have yielded favorable surgical outcomes in TOS [25–28]. These approaches provide excellent visualization of the entire first rib, which can be resected posteriorly up to the costotransverse joint; however, they require pleural drain insertion in all cases [26, 28]. Additionally, the described surgical approach is feasible even in cases involving pleural adhesions or intolerance to one-lung ventilation, which are considered contraindications for intrathoracic techniques. Furthermore, the infraclavicular approach enables direct exposure and dissection of the subclavian vein within a shallower field, facilitating safe rib resection with subclavian vein retraction, venolysis, and reconstruction [4, 5], which may offer significant advantages in venous TOS surgery.

This study had four major limitations. First, due to the small sample size and retrospective nature of the study, this study could be subject to selection bias, which reduces its generalizability of the outcome of AR-assisted IC-FRR. Second, because this was a nonrandomized retrospective study without a control group, we could not compare the outcomes without the use of AR technology. Third, long-term outcomes were not assessed. Fourth, this study did not include a 3D accuracy assessment of the extent of rib resection using postoperative CT images; therefore, the accuracy of the AR system could not be evaluated. Accuracy may be influenced by potential sources of error, including cumulative deviations in position between the bone and the AR marker, as well as display-related inaccuracies in the AR system. Enhancing the contact surface area between the AR marker–mounted block and the clavicle may reduce positional deviation errors and improve overall accuracy.

The described technique employs an AR marker affixed to the clavicle as a reference point to delineate the anatomical positions of adjacent blood vessels and osseous structures. This technique may have additional applications in procedures involving the subclavian arteries and veins, such as subclavian artery-to-upper limb artery bypass surgery and subclavian vein reconstruction surgery, as well as surgeries for bone tumors occurring in the sternum or ribs.

In summary, this study provides new insights into IC-FRR for vascular TOS. AR technology assistance enables intraoperative 3D assessment of the location of major blood vessels and the extent of FRR, which may contribute to improved postoperative outcomes.

## Data Availability

Data supporting the findings of this study are available from the corresponding author upon reasonable request.

## Abbreviations

AR: Augmented reality
CT: Computed tomography
DASH: Disability of the arm, shoulder, and hand
FRR: First rib resection
IC-FRR: Infraclavicular first rib resection
TOS: Thoracic outlet syndrome
3D: Three-dimensional
